# Newly identified intervertebral fat pad degenerates after intervertebral disc injury in a rat model of degeneration

**DOI:** 10.1111/joa.70092

**Published:** 2025-12-14

**Authors:** Niklas H. Koehne, Irina Heggli, Agnieszka A. Karol, Alon Lai, Svenja Illien‐Jünger, Andrew C. Hecht, Nilsson Holguin, James C. Iatridis

**Affiliations:** ^1^ Leni & Peter W. May Department of Orthopedics Icahn School of Medicine at Mount Sinai New York City New York USA; ^2^ Musculoskeletal Research Unit (MSRU), Department of Molecular Mechanisms of Disease (DMMD), Vetsuisse Faculty University of Zurich Zurich Switzerland; ^3^ Emory Musculoskeletal Research Center, Department of Orthopaedics Emory University School of Medicine Atlanta Georgia Atlanta Georgia USA; ^4^ Wallace H. Coulter Department of Biomedical Engineering Georgia Institute of Technology Atlanta Georgia USA; ^5^ Joseph Maxwell Cleland Atlanta VA Medical Center Decatur Georgia USA

**Keywords:** disc degeneration, human disc degeneration, intervertebral disc, intervertebral fat pad, IVD, mouse disc degeneration, puncture‐induced intervertebral disc degeneration, rat model

## Abstract

Chronic low back pain due to intervertebral disc (IVD) degeneration (IVDD) is a major global health burden. Interactions between IVD tissues and surrounding structures are important for spinal health and pathology, yet many studies focus on structures within the IVD and neglect a deeper investigation of adjacent tissues. This study describes a newly identified intervertebral fat pad (IVFP) in rat lumbar spines, its changes following IVD injury, and similar structures in mice and humans. IVFPs were analyzed histologically using naive and injured IVDs from a rat model of IVDD, in which 5‐month‐old rats underwent a triple‐puncture annulus fibrosus (AF) injury of L3‐4, L4‐5, and L5‐6 IVDs. Sagittal and coronal histologic samples were stained with safranin O/fast green and analyzed at 3, 7, 14, and 56 days post‐injury. Naive and sham IVDs demonstrated the consistent presence of an IVFP between the anterior AF and anterior longitudinal ligament (ALL) in anterior and anterolateral regions of the IVD, without presence at posterior or postero‐lateral IVD regions. The IVFP gradually disappeared in injured IVDs, and became largely absent by 56 days post‐injury. Post‐injury changes to the IVFP also included adipocyte shrinkage, fibrous tissue infiltration, and gradual IVFP disappearance, together suggesting progressive degeneration. IVFP‐like structures were identified histologically in mouse and human IVDs, providing evidence of its presence across species. Fat pads studied in other musculoskeletal joints play roles in health and disease, suggesting a need for further study investigating the potential role of the IVFP in IVDD pathomechanisms and therapeutics.

## Graphical abstract

This study characterizes a newly‐described intervertebral fat pad (IVFP) anterior to rat IVDs, with evidence for its existence in mice and human IVDs as well. In rats, the IVFP gradually degenerated following IVD injury with changes in adipocyte size and fibrous tissue infiltration, suggesting it could play a functional role in IVD health and degeneration.

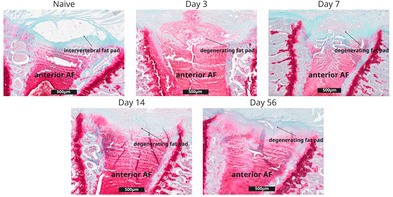

## 1 INTRODUCTION

Chronic low back pain is a major burden on global health and a leading cause of chronic disability in adults. It is estimated that chronic low back pain affects 70–85% of the population at some point in their lives and reports an economic burden of nearly 140 billion dollars in 2016 (de Souza et al., 2019; Vos et al., 2012). Chronic low back pain can result from many pathologic mechanisms, including degeneration of intervertebral discs (IVDs) and facets, stenosis, and spinal fibromyalgia (Li et al., 2021). Among these, IVD degeneration (IVDD) is a leading underlying pathology and is the most important risk factor for developing chronic low back pain and disability (Fujii et al., 2019; Livshits et al., 2011). Despite the prevalence of IVDD, surgical and medical treatments have limited efficacy, demonstrating the continued need for basic science research that more fully elucidates IVDD phenotypes and pathogenesis (Nicol et al., 2023).

Anatomic fat pads have been characterized in the bodies of rats and humans and are known to serve essential roles in joints, yet their description in the context of back pain and IVDD remains limited (Raza et al., 2024). In humans, the infrapatellar fat pad is a morpho‐functional component of the knee, providing joint cushioning, multiple endocrine, and inflammatory cytokines that can regulate joint physiology and disease (Macchi et al., 2018; Tang et al., 2024; Wang et al., 2024). Furthermore, the infrapatellar fat pad significantly modulates the inflammatory response in knee osteoarthritis, and some clinical results suggest its partial resection in osteoarthritis yields favorable outcomes (Li et al., 2025; Park et al., 2025; Xu et al., 2025). While spinal joints have several anatomical parallels with knee joints, fat pad structures are not described in the spine. However, fatty changes via obesity have been associated with IVDD in humans and animal models (Robbins et al., 1989; Samartzis et al., 2011; Taylor & McCormick, 1991). Additionally, adipokines in the spine are associated with IVDD and pro‐inflammatory cytokines, suggesting some phenotypes of IVDD and back pain could be fat‐related conditions (Samartzis et al., 2013).

Animal models can be applied to study IVDD progression and extra‐discal tissues such as fat pads at multiple time points post‐injury, providing data such as pain‐like behaviors, imaging, and post‐mortem tissue analysis with gene, protein, cellular, and histological focuses. Rodents are common preclinical models for IVDD due to their structural similarity to human spines, quantifiable pain metrics, and ability to undergo controlled research studies (Alini et al., 2023; Shi et al., 2018; Tang et al., 2022). Histological analyses can identify structural, cellular, and matrix changes across different stages of life, including pathological alterations, cellular infiltration, morphological changes, inflammatory processes, neovascularization, and tissue repair. Histology remains a gold standard for the diagnosis of many diseases, and can accurately differentiate pathologic mechanisms that may result in similar disease phenotypes.

In the context of IVDD, many histological methods can be applied to characterize disease progression and injury responses over time (Walter et al., 2015). Histology of the spine most commonly focuses on IVD regions and commonly discards soft tissues anterior and posterior to the IVD that may play functional roles as a source of cells, cytokines, and metabolic factors (Mosley et al., 2017). Histology and immunohistochemistry approaches also include detailed descriptions of cells and tissues, which necessarily involve highly magnified investigations of small regions of interest. More traditional histological methods and microscopy imaging modalities commonly exclude extraneous tissue to simplify fixation methods and keep image file sizes smaller. Slide scanners have made the analysis of larger specimens more efficient, allowing for a more feasible exploration of extra‐spinal tissues and the identification of new structures such as fat pads surrounding the spine.

Despite the growing evidence that fat pads are important anatomical structures in multiple joints, very little is known about fatty tissues surrounding IVDs. In this study, we identified an intervertebral fat pad (IVFP) anterior to rat IVDs and could not find literature describing this new anatomic feature. We also observed that the IVFP disappeared following puncture‐induced IVD degeneration, suggesting this structure is a potential player in spinal pathology. Therefore, this study aimed to (1) describe anatomic features of the IVFP in healthy and injured IVDs in a rat IVDD model, (2) describe how the IVFP degenerates following AF puncture injury, and (3) evaluate IVDs of mice and humans for the presence of an IVFP.

## 2 METHODS

### 2.1 Study design and rat model

Naive, sham‐injured, and injured IVDs from a previously established rat model of IVDD were analyzed. Skeletally mature male Sprague–Dawley rats aged 4–5 months old were used, and all experimental procedures were approved by the Institutional Animal Care and Use Committee (Evashwick‐Rogler et al., 2018; Lai et al., 2024; Wang et al., 2023). Naive rats did not undergo any surgical procedure. In injured rats, IVDD was induced by a triple‐puncture injury through the anterior annulus fibrosus (AF). Briefly, the anterior aspect of the lumbar spine was exposed using an abdominal incision through the skin and peritoneal membrane. After identifying the L3‐4, L4‐5, and L5‐6 IVDs, each IVD was punctured three times (i.e., midline anteriorly as well as left and right anterolaterally) using a 26G needle at a depth of 3 mm guided by a needle stopper including needle twisting and side‐to‐side motion, followed by an intradiscal injection of sterile PBS using a calibrated microliter syringe (Hamilton Company, Reno, NV, USA). Sham surgery exposed L3‐4, L4‐5, and L5‐6 IVDs only and did not include needle puncture into IVDs. Surgeries were performed under aseptic conditions with general anesthesia via 2% isoflurane. It should be noted that the tissues anterior to the IVD were carefully displaced during sham and injury surgical procedures, and puncture injury was focused on the IVD with little perturbation of surrounding tissue. After injury, rats were euthanized at 3, 7, 14, and 56 days post‐injury (dpi) to capture both acute and chronic injury responses. Sham data came from a previous cohort of rats collected at 42dpi only (*n* = 7); however, this sham procedure was designed as a control for the same puncture injury used in this study (Evashwick‐Rogler et al., 2018). All animals were euthanized with transcardial perfusion of 10% buffered formalin phosphate (Fisher Company, Fair Lawn, NJ, USA) under anesthesia. The L4‐6 lumbar spines were isolated while retaining surrounding soft tissues and further fixed in acetic zinc formalin for at least 48 h, as described (Lai et al., 2024).

**FIGURE 1:**
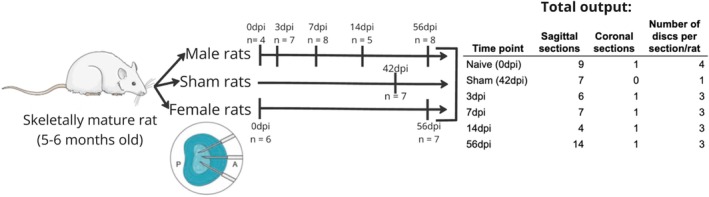
Overview of rat samples from naive, sham, and annulus fibrosus (AF)‐injury surgeries. Spine sections from each group were collected at the time points indicated on the timeline. Naive rats were classified as 0 days post‐injury (dpi) because they had not undergone any surgical intervention and represented the pre‐surgical baseline. For statistical comparisons, sham rats collected at 42 dpi were compared to AF injury groups collected at 56 dpi.

Male rats selected for this study included 4 naive rats (3 sagittal, 1 coronal), 7 sham rats (7 sagittal), 7 rats at 3dpi (6 sagittal, 1 coronal), 8 rats at 7dpi (7 sagittal, 1 coronal), 5 rats at 14dpi (4 sagittal, 1 coronal), and 8 rats at 56dpi (7 sagittal, 1 coronal). Sample sizes used for quantification only included sagittal sections (12 naive IVDs, 7 sham IVDs at 42 dpi, 18 injured IVDs at 3dpi, 21 injured IVDs at 7dpi, 12 injured IVDs at 14dpi, and 21 injured IVDs at 56dpi) (Figure 1).

Female rat samples of naive (*n* = 6 rats, 24 IVDs) and 56dpi (*n* = 7 rats, 21 IVDs) were included for a sex comparison of fat pad morphology and its changes in response to injury. Female samples were processed the same way as male samples but as a separate cohort (Figure 1).

### 2.2 Sample preparation, staining, and imaging

Formalin‐fixed spine specimens were decalcified in formic acid over 3 days with 3 changes, embedded in paraffin, and sectioned sagittally or coronally at 5 μm intervals. Midsagittal and mid‐coronal sections were identified by comparing the relative sizes of the AF and nucleus pulposus and identifying needle tracks from punctures done at the midsagittal plane when possible. Sections were then stained with safranin O/fast green/hematoxylin for visualizing GAG content, IVD morphology, and IVD cellularity (Lai et al., 2024). The combination of Safranin O and fast green stains is a well‐established histologic methodology and allows for clear differentiation of spinal tissues at and around the IVD (Lai et al., 2021). The stained slides were imaged using a digital slide scanner (C13210‐01 NanoZoomer S60, Hamamatsu Corporation, Bridgewater, NJ, USA).

### 2.3 Human and mouse samples

Human and mouse samples were taken from previously published studies with clear sagittal sectioning of lumbar IVDs (Nakazawa et al., 2018; Walter et al., 2015). Human samples included 23 sagittal lumbar IVDs from male and female specimens (48% male), ranging from 4 months old to 93 years old (median = 53 years). Mouse samples included 10 sagittal lumbar IVDs from male and female mice (50% male) aged 9 months to 18 months that were fed normal chow as well as low and high advanced glycation endproduct chow (Illien‐Junger et al., 2013; Illien‐Jünger et al., 2015).

### 2.4 IVFP analysis and quantification

**FIGURE 2:**
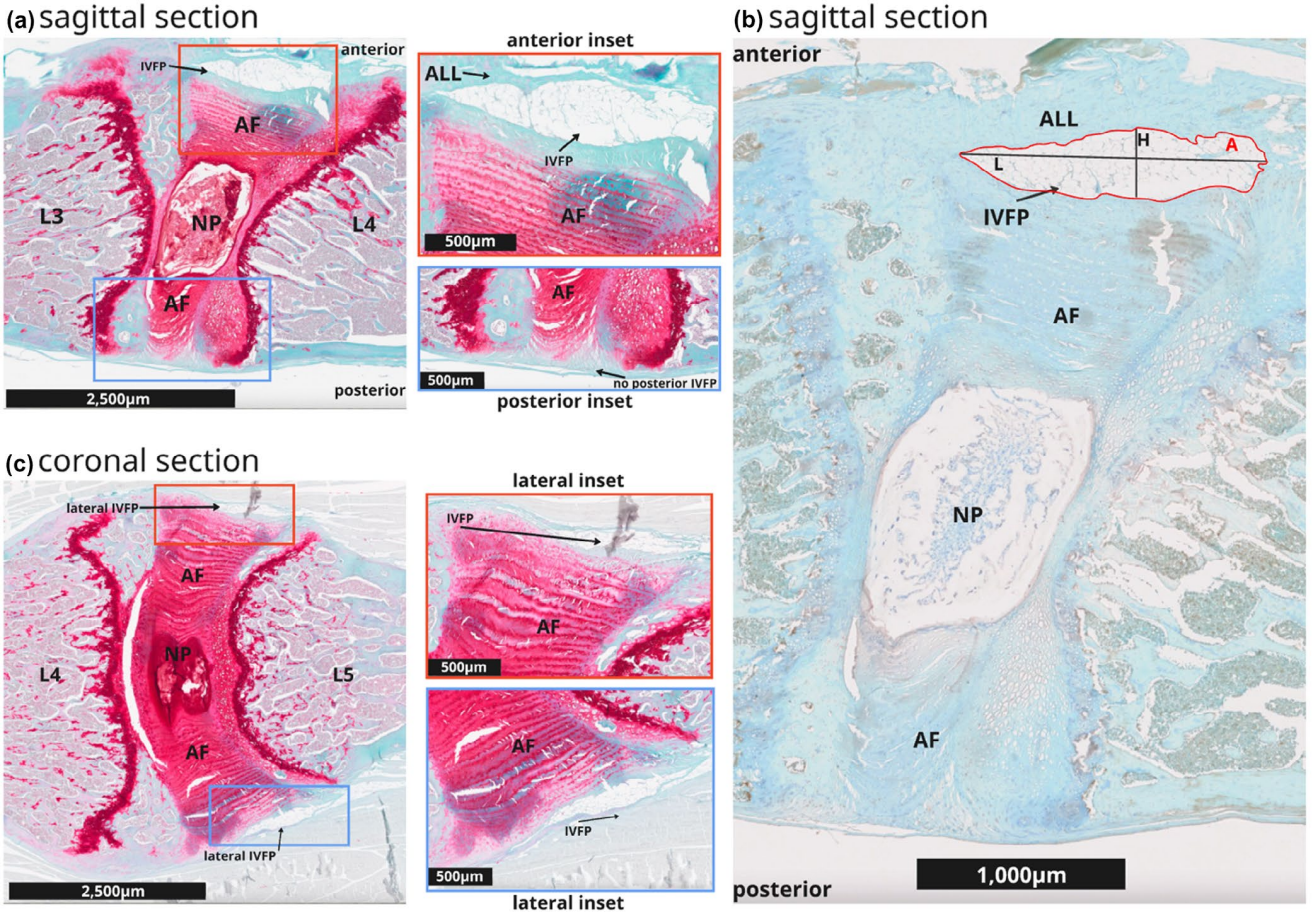
Intervertebral fat pad (IVFP) analysis. (a) Representative Safranin O/Fast Green–stained image of a naive anterior IVFP, composed of homogeneous adipose tissue and bordered anteriorly by the anterior longitudinal ligament (ALL) and posteriorly by the anterior annulus fibrosus (AF). The image shows an L3–4 sagittal section centered on the nucleus pulposus (NP). (b) L3–4 sagittal section stained with toluidine blue, illustrating area (A), height (H), and length (L) measurements. (c) Coronal section of L4–5 demonstrating lateral extension of the IVFP, with a tapering width. Scale bars for overview images represent 2.5 mm, and scale bars for magnified images represent 500 μm.

The presence of the IVFP and its anatomic borders was verified by pathologists in naive and sham rats, and a region of interest for all IVDs was identified. In naive rats, several dimensions of the IVFP were measured using ImageJ software, including 2‐dimensional area on midsagittal section, maximum height, and maximum width (Figure 2). Total IVD height was also measured from the anterior side of the anterior longitudinal ligament (ALL) to the posterior side of the posterior longitudinal ligament (PLL) and was used to calculate relative IVFP height (IVFP height / total IVD height). In injured rats, the disappearance of the IVFP was evaluated for changes following IVD injury by quantifying the percentage of fat remaining, accounting for any fibrotic or proteoglycan infiltrate that may have replaced adipose tissue. This grading scale was manually estimated and ranged from 0% to 100%, with a score of 100% describing a complete IVFP without shrinkage, border degradation, or fibrotic changes. An IVFP scored at 50% described a fat pad that was roughly half the size of a healthy IVFP, often accompanied by infiltration of inflammatory cells, degradation of the IVFP's borders, and splitting of fat lobules. Finally, a score of 0% described an IVFP that fully disappeared, meaning no adipose tissue could be visualized. These samples presented with dense fibrotic tissue that has replaced the IVFP. IVFPs were observed to be similar across animals and dependent on local injury. Therefore, IVFPs were graded individually and treated as their own replicate such that each rat yielded 3 IVFP values, corresponding to the L3‐4, L4‐5, and L5‐6 levels. Each IVFP was evaluated by two graders, and scores were averaged to produce a single value per rat. In the case of >10% inter‐reviewer differences (18.5% of the cases), morphological features of the IVFP were discussed between graders, followed by a re‐scoring of the IVFP by each grader. Sagittal sections were included for quantification due to sample size and consistency of the sectioned plane. Coronal sections were assessed qualitatively to determine anatomical and lateral boundaries of the IVFP.

### 2.5 Statistical analysis

The percent fat pad remaining in the naive and sham IVFPs was tested for normality using the Shapiro–Wilk test. Since these groups were nonparametric, a Mann–Whitney U test was performed to compare the differences between the two groups. The Shapiro–Wilk test was also used to test for normal distribution in injured samples. The data were then tested for outliers using the Robust Regression and Outlier Removal (ROUT) test. Based on this analysis, one outlier at 56 dpi was removed. A parametric one‐way ANOVA was performed to analyze the percent presence of fat pads over time, followed by Tukey's post hoc pairwise comparisons. A Shapiro–Wilk test and subsequent Mann–Whitney U test were also used to compare the male and female groups. All analyses and data visualization were done in GraphPad Prism version 10.

## 3 RESULTS

### 3.1 IVFP anatomical location and size in uninjured rat IVDs

An IVFP was identified in all naive IVDs at an anatomical location anterior to the IVD, bounded by the anterior AF and ALL (Figure 2a,b). The IVFP also extended laterally towards the center of the IVD, demonstrated by its presence at the anterolateral sides of the IVD when evaluated on coronal sections exhibiting nucleus pulposus tissues (Figure 2c). On sagittal view, the IVFP measured between 200 and 400 μm thick at its widest portion (at the mid‐transverse plane), and tapered laterally to 100–125 μm thick as it approached the superior and inferior vertebral endplates. Maximum IVFP height and its height relative to IVD height were significantly larger in male rats than female rats; however, IVFP length and two‐dimensional area did not differ significantly between the sexes (Table 1). Healthy IVFPs, present in uninjured naive IVDs, were comprised of homogenous adipose tissue that remained contained by well‐demarcated borders of fibrous tissue. There was no evidence for an IVFP at posterior or postero‐lateral regions of the IVD, as the posterior longitudinal ligament ran directly adjacent to the posterior AF. Sham animals also demonstrated a largely uninjured fat pad, with either no or minimal degeneration of the adipose tissue and its borders (Figure S1). There was no significant difference in the percentage of fat pad remaining between naive and sham IVDs (94.2% ± 3.74% vs. 87.1% ± 13.8%, respectively; Mann–Whitney U test, *p* = 0.37). Equivalency between naive and sham groups demonstrated that perturbation of extra‐spinal tissue during surgery did not confound IVFP due to puncture.

**TABLE 1:** Summary of intervertebral fat pad (IVFP) dimensions in naive male (*n* = 4) and female (*n* = 6) rats.

	IVFP height (μm)	IVFP length (μm)	IVFP area (μm^2^)	IVFP relative height
Male rats	356 ± 70	1367 ± 317	343 ± 114	0.103 ± 0.023
Female rats	236 ± 70	1334 ± 321	215 ± 120	0.067 ± 0.020
M vs. F significance	*p* < 0.001	*p* = 0.542	*p* = 0.088	*p* = 0.0012

*Note*: Values are reported as mean ± standard deviation (SD). Relative IVFP height was calculated by dividing the IVFP height by the total intervertebral disc height.

### 3.2 IVFP disappeared over time after AF puncture injury

**FIGURE 3:**
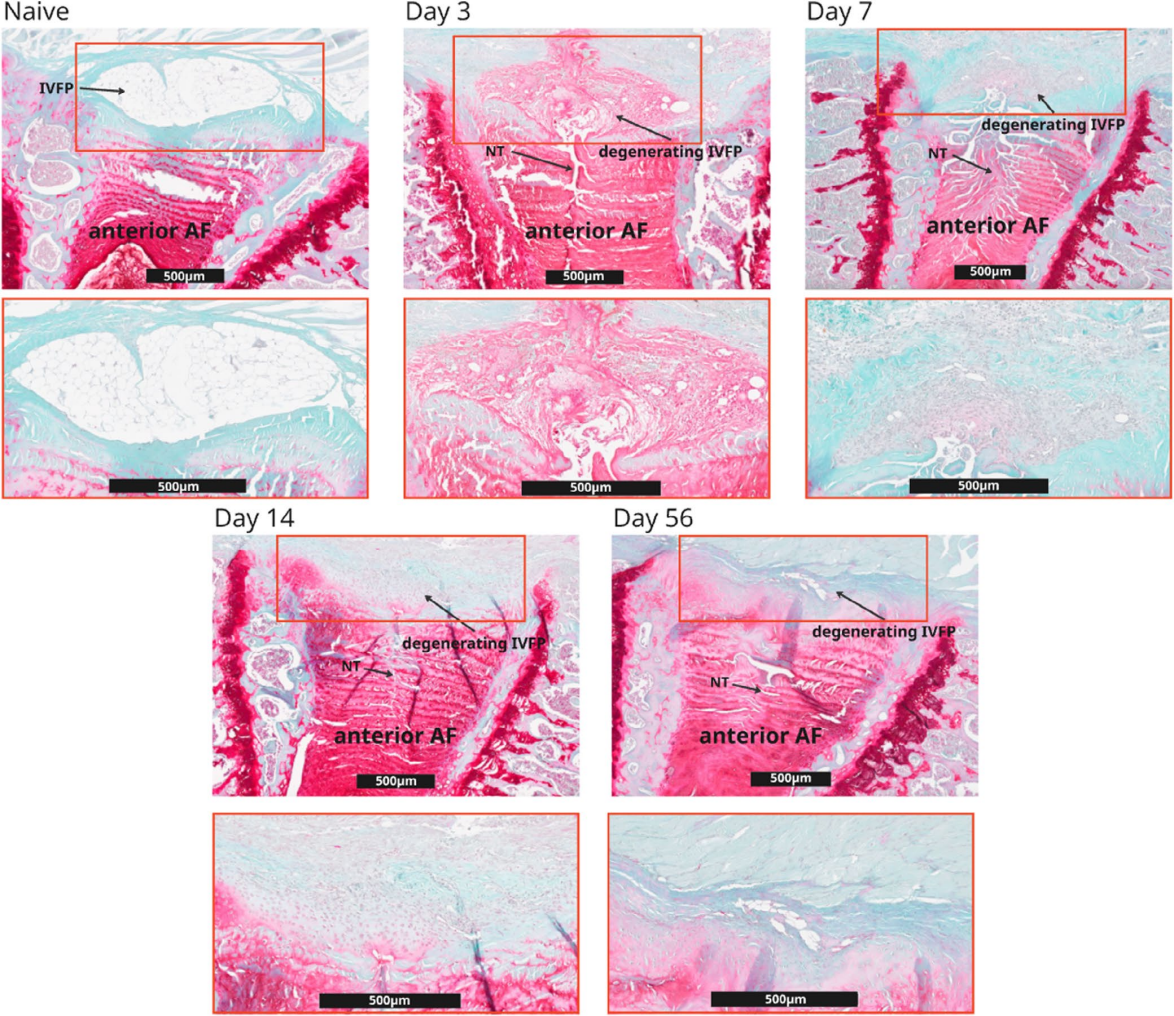
Histology of injured intervertebral fat pads (IVFPs) over time. Representative IVFP histology is shown for all time points. Scale bars represent 500 μm, and all images are oriented with the anterior annulus positioned at the top of the page. Sample sizes were naive (*n* = 12), day 3 (*n* = 18), day 7 (*n* = 21), day 14 (*n* = 12), and day 56 (*n* = 21), where *n* corresponds to the number of intervertebral discs examined. Residual needle tracks (NTs), when present, are labeled to indicate the precise entry site of the surgical needle.

**FIGURE 4:**
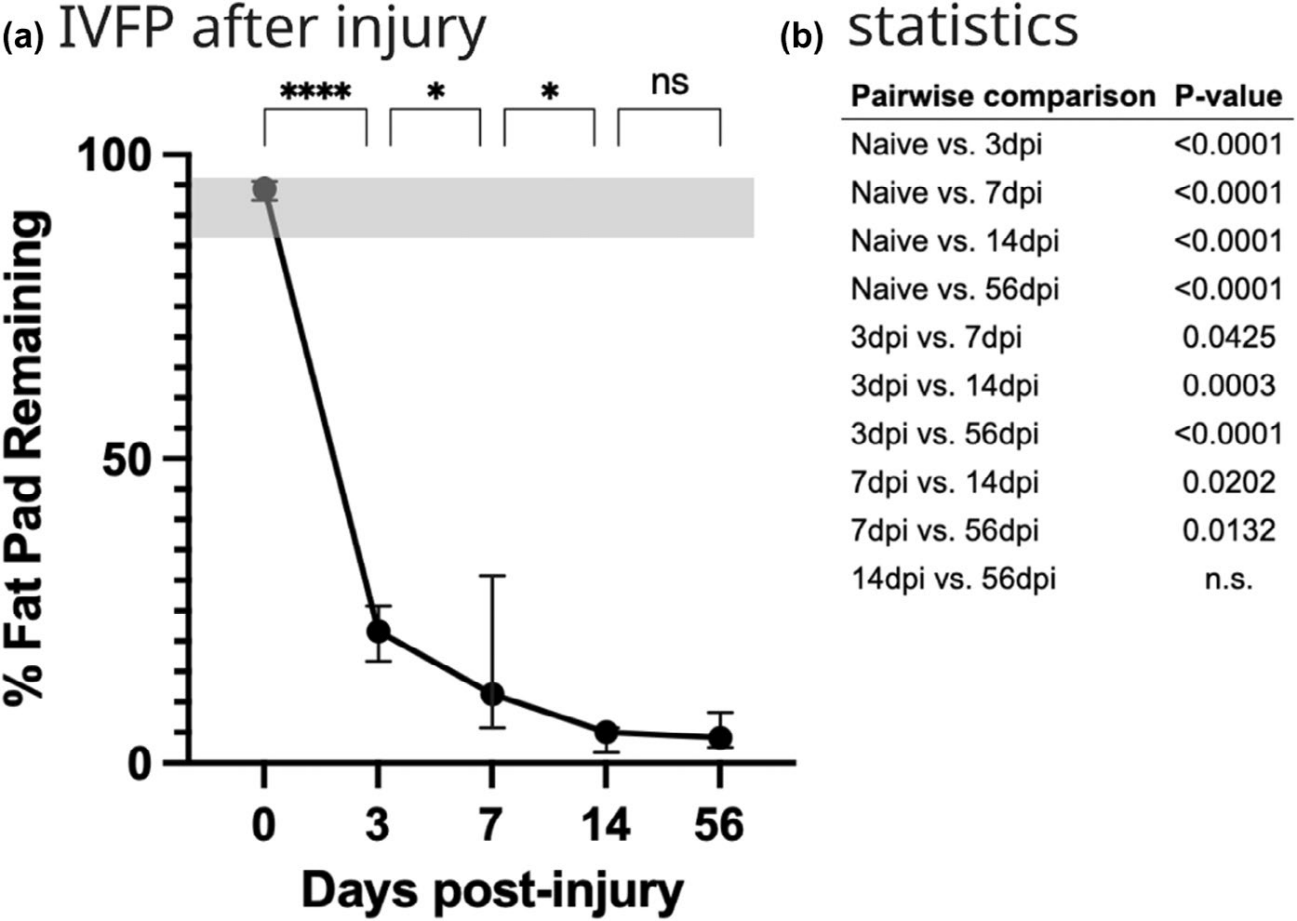
Intervertebral fat pad (IVFP) disappearance over time. (a) IVFP percent presence declined progressively after AF puncture injury, with a significant reduction observed as early as 3 days post‐injury (dpi). All post‐injury time points were compared with naive controls. (b) Percent IVFP presence decreased over time, with mean ± SD values of 21.7 ± 3.2% at 3 dpi, 14.3 ± 9.1% at 7 dpi, 4.4 ± 1.9% at 14 dpi, and 4.6 ± 2.3% at 56 dpi. The gray bar indicates the percent fat pad remaining in sham rats at 42 dpi (87.1 ± 13.8%).

After AF puncture injury, IVFPs in male rats showed a progressive loss of fat pad tissue (Figure 3). By 3 dpi, a significant reduction was already evident, with only 21.7% ± 3.2% of the fat pad remaining (Figure 4a). Reduction in fat content continued throughout 7dpi (mean = 14.3% ± 9.1%), 14dpi (mean = 4.4% ± 1.9%) and was nearly complete by 56dpi (mean = 4.6% ± 2.3%). At 56dpi, females also showed near complete reduction of the IVFP (mean = 5.4% ± 3.14% of fat pad remaining). The disappearance observed in males vs. females was not statistically different at 56dpi (*p* = 0.57). The pairwise comparison (Figure 4b) further demonstrated significant disappearance compared to earlier time points in male rats.

### 3.3 Extracellular matrix and lipid droplet changes

**FIGURE 5:**
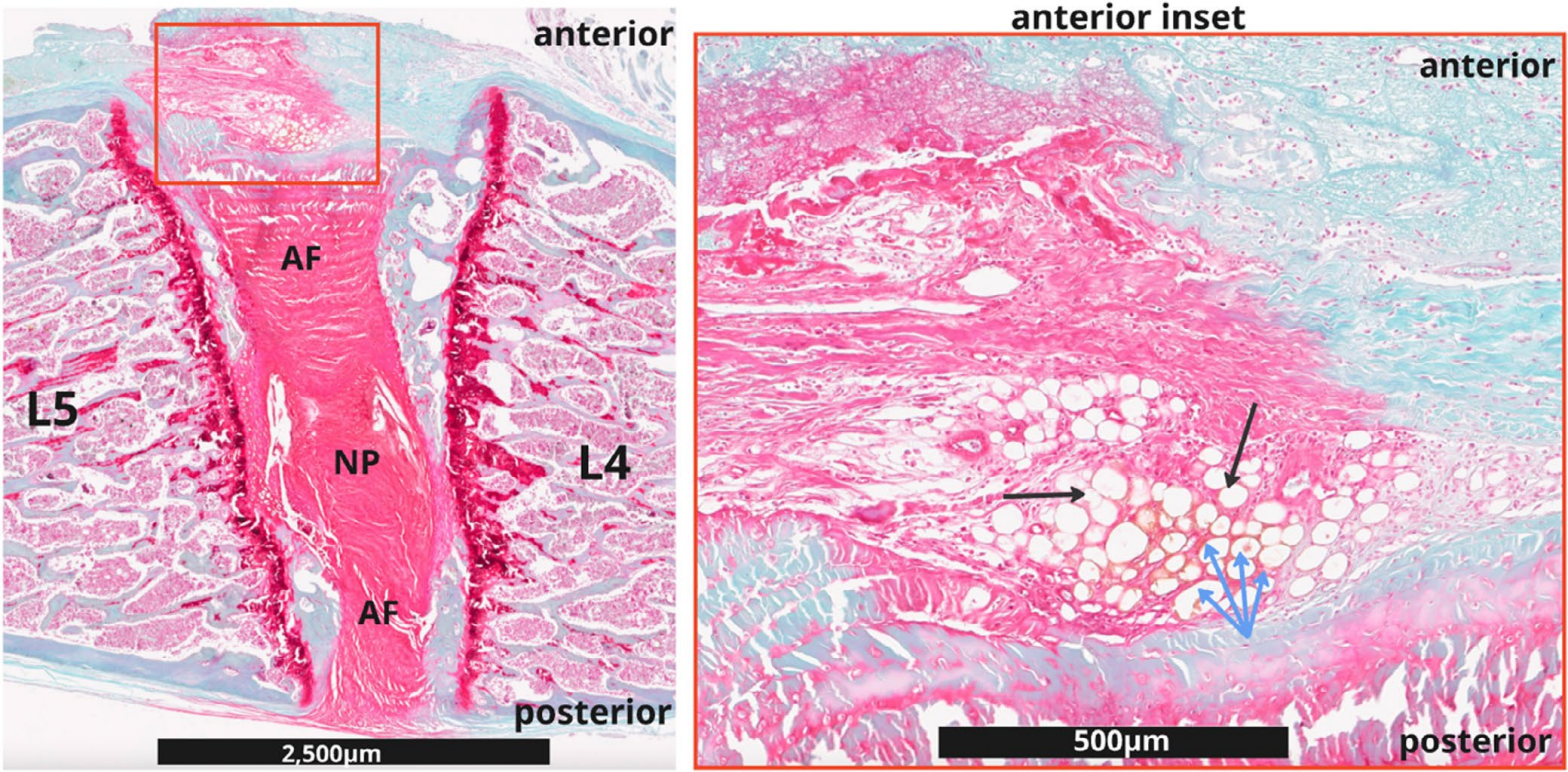
Intervertebral fat pad (IVFP) cellular changes. Representative histology of the IVFP 3 days post‐injury, demonstrating separation of fat lobules (blue arrows) and fibrous tissue infiltration (black arrows). Scale bar represents 500 μm.

IVD injury disrupted the clear margins demarcating the IVFP and its surrounding ALL and anterior AF borders (Figure 5). Additionally, the IVFP matrix shifted from lipid droplets to a more fibrous structure with increased safranin O staining within the IVFP, indicating the presence of a proteoglycan‐rich matrix. Lipid droplets became smaller, seemed to increase in number, and became surrounded by fibrous infiltration as the lobules separated (Figure 5). At later time points (14dpi and 56dpi), a more homogenous collagenous tissue dominated the IVFP region (Figure 3).

### 3.4 Existence across species

**FIGURE 6:**
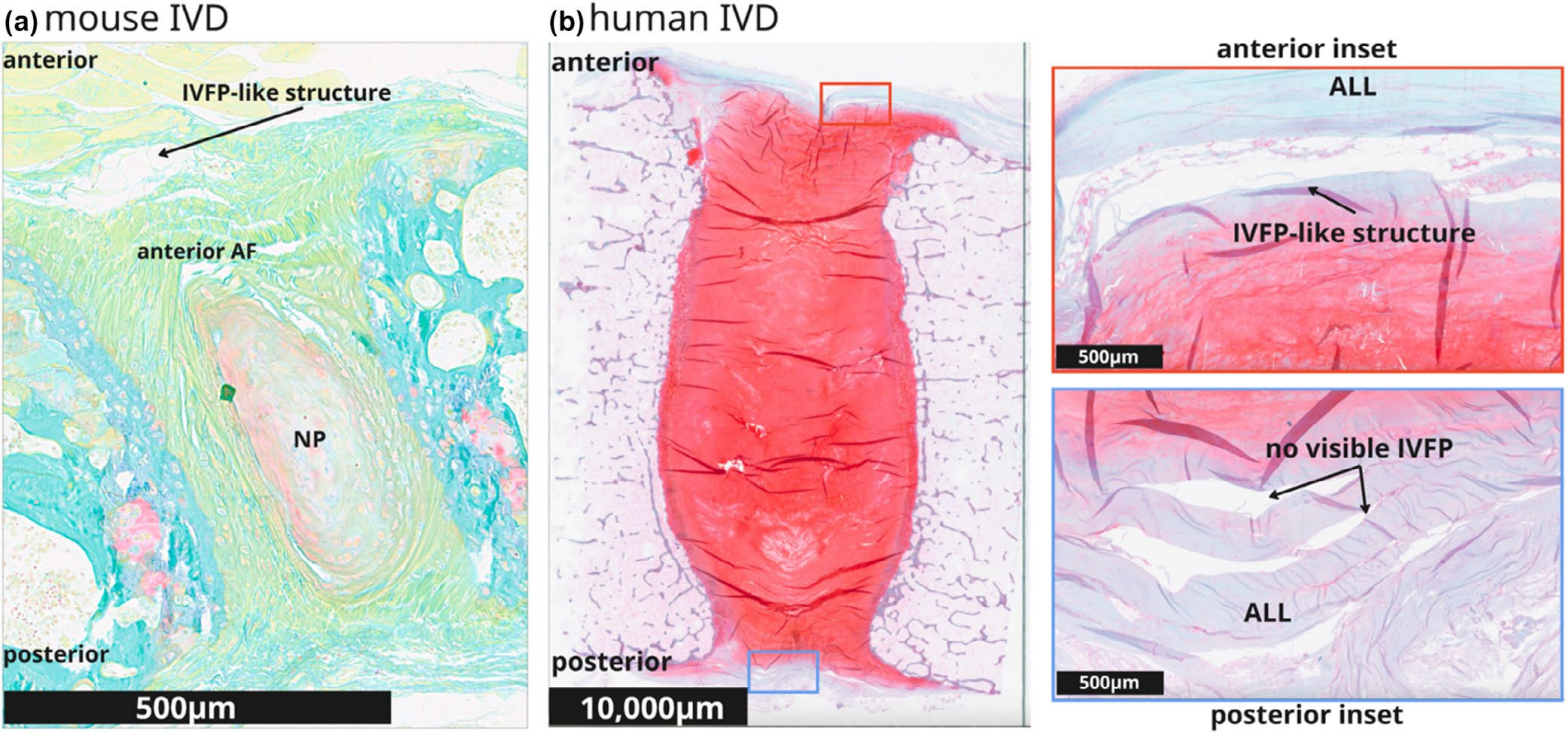
Mouse and human intervertebral fat pads (IVFPs). Evidence of an anterior IVFP in: (a) sagittal section of lumbar IVD from 4‐month‐old mouse, (b) sagittal section of human L3‐4 IVD from 66‐year‐old female.

Histological sagittal sections of human and mouse IVDs were investigated to assess the presence of an IVFP structure in different species. An anterior IVFP with the same anterior AF and ALL anatomic borders was observed in naive mouse lumbar IVDs (Figure 6a). On midsagittal sectioning, mouse IVFPs had an average height of 48.1 μm ± 14.2 μm and an average relative height of 0.046 μm ± 0.022 μm. In human IVDs, an anterior IVFP was also identified between the outer AF and the ALL (Figure 6b). Of the 23 human IVDs reviewed, 5 (4 female, 1 male) demonstrated potential evidence of a fat pad across a wide range of ages, and 13 were deemed inconclusive due to a lack of tissue anterior to the IVD. Among samples with visible fat pads, human IVFPs had an average height of 0.44 mm ± 0.10 mm and an average relative height of 0.014 mm ± 0.003 mm.

## 4 DISCUSSION

This study describes a previously uncharacterized IVFP composed of homogeneous fat tissue bordered by the anterior AF and ALL, and presents in rats, mice, and human spinal structures. The IVFP extends laterally but does not reach posterior IVD regions. Only AF puncture injury (not Sham) caused the IVFP to gradually disappear, including breakdown of fat lobules and fibrous tissue infiltration by 3 days post‐injury, suggesting IVFP changes may play a role in injury response. By 56 dpi, the IVFP remained significantly reduced and did not show signs of regeneration. The IVFP was similar in naive and sham groups, indicating that IVFP degeneration was dominantly driven by AF puncture‐induced IVDD. Given fat pads are known to play important metabolic roles in multiple joints throughout the body, this paper highlights the presence of an IVFP and supports future investigations to determine potential biological and clinical significance.

The anatomy of the IVFP suggests it could play a mechanical role in rat spines, such as padding, or allowing micromotions between the anterior AF and ALL. For example, the infrapatellar fat pad is thought to cushion and lubricate the knee joint, both by leveraging the slippery texture of fat tissue and by actively secreting lubricating proteins such as lubricin (Theobald, 2013). Fat pads are also effective at absorbing blunt forces, such as the sub‐calcaneal fat pad, which plays a critical role in protecting the plantar surface of the foot from fracture and ulceration (Bus et al., 2021). The IVFP may similarly play a mechanical role at the anterior spine, and its loss with IVDD could therefore modulate tissue strains on the IVD and contribute to altered motion of pain with movement. Alternatively, the fat pad may also play a role in stabilizing the IVD by filling gaps between joint tissues (Zeng et al., 2020). It may be possible that the lack of an IVFP in posterior regions contributes to the prevalence of posterior herniations compared to herniations in other anatomic directions. Finally, we cannot exclude the possibility that ALL injury contributes to IVFP degeneration, although ALL disruption was minimized during puncture injury and remained very limited in sham procedures.

The disappearance of the IVFP after injury suggests it may also play a metabolic role in IVDD progression. There are several potential explanations for the disappearance of the IVFP post‐injury, including direct destruction during anterior puncture surgery, local inflammation causing degradation by infiltrating immune cells, reduced oxygen supply due to disruption of surrounding vasculature, and/or dedifferentiation of adipocytes into fibroblasts as part of a healing response (Favero et al., 2024; Grigoraș et al., 2021; Liao et al., 2015). Additionally, injury‐induced alterations in spinal mechanics may alter loads applied to the IVFP during movement, causing potential fat disappearance via increased pressure or friction. Of these possibilities, direct destruction by puncture surgery is less likely, primarily because no significant reduction of the IVFP was observed in sham animals and changes to the fat pad continued to progress up to 2–8 weeks after injury. These delayed changes suggest that some components of IVDD progression may be linked to IVFP disappearance.

Metabolically, adipocytes in the IVFP could release cytokines and chemotactic agents that stimulate their own digestion via phagocyte recruitment. By analogy, perirenal fat pads harbor mesenchymal stem cells and can secrete interleukins such as IL‐6 and IL‐8 as part of an inflammatory response (Grigoraș et al., 2021). If the IVFP similarly harbors mesenchymal stem cells or secretes inflammatory cytokines, then it is possible that the fat pad itself could be involved in the homing of phagocytes to the site of injury. The IVFP could therefore be a contributor of cytokines that drive immunologic remodeling or painful conditions.

Adipocytes within damaged fat pads have been shown to dedifferentiate into fibroblasts (Favero et al., 2024; Liao et al., 2015). It is therefore interesting to consider that IVFP disappearance post‐injury could be a remodeling process involved in fibrous scarring. In this study, the IVFP demonstrated metabolic changes involving dedifferentiation of adipocytes post‐injury. The splitting of lipid droplets and fibrous tissue infiltration observed in this study was previously reported as evidence of adipocyte dedifferentiation into fibroblasts as part of a stress response (Liao et al., 2015). By 56 dpi, IVFPs were largely replaced by connective tissue in this study, also supporting the possibility that adipocyte disappearance is related to dedifferentiation into ECM‐secreting fibroblasts.

Evidence of an IVFP was also observed in mice and humans. In 4‐month‐old lumbar mouse spines, a fat pad structure very similar to those found in rats was observed, sharing both anatomic boundaries and adipocyte composition, but with smaller relative size. In humans, the IVFP demonstrated similar boundaries and composition, but was proportionally thinner compared to rat IVFPs. The fat pad's smaller size in humans may be due to the fact that human cadaveric images observed were from older adults and therefore may have partially degenerated throughout a lifetime. The small size of this fat pad in humans also illustrates that such a structure could easily be missed grossly and on MRIs, and thus requires a high‐resolution histologic examination of spines with anterior tissue maintained. Furthermore, the large human IVD size means it is common to “clean” surrounding tissues away from the IVD to better allow penetration of fixation and embedding materials, providing another reason why IVFPs have not been described in human IVDs. However, a thinner IVFP may also suggest a less prominent role in human IVDs.

### 4.1 Limitations

The main contribution of this study is to highlight a newly described IVFP structure that disappears post‐injury and could play functional roles in IVD health and IVDD progression. We focused on male and female rats 5–6 months old, with less systematic evaluations of available mouse and human cohorts amenable to characterizing IVFP structures. Injured IVFPs differed significantly from naive and sham samples, demonstrating that puncture injury triggered fat pad disappearance. Certain limitations of these results exist. Sham data was taken from a previous article published in 2018, which consisted of the same puncture injury used in this study but included a small sample size with good IVFP visualization, possibly leading to the high variance observed in the sham group (Evashwick‐Rogler et al., 2018). Mouse and human samples were also taken from previous data sets, with many of these samples not containing sufficient tissue anterior to the IVD for proper IVFP analysis. Thus, future studies investigating the IVFP across species, age, spinal level, and sex, with sample preparation geared for fat pad visualization, are needed to clarify the generalizability of the IVFP, although it is notable that an IVFP can be identified in histological samples across the IVD literature even though this anatomical structure is not previously described (Lillyman et al., 2022; Mosley et al., 2019). Future research may also further explore the potential mechanistic roles of the IVFP proposed in this study. Spinal ligaments and vertebrae are vital for spinal stability and have been well characterized across species, though often without a detailed histologic analysis (Belyaev & Prilepskaya, 2025; Hennigan et al., 2025). Studies that continue this anatomic work may benefit from additional histology focused on the IVD and surrounding tissues that may not appear on radiographic imaging. Further research applying histological based approaches to screen the IVFP with small trims throughout the entire width of the sagittal spine would be helpful to create a full volume view of the size and shape of the IVFP.

## 5 CONCLUSION

This study identified a new intervertebral fat pad anterior to the IVDs of rats, with possible existence in mice and human IVDs as well. The IVFP disappeared gradually over 56 days following IVD injury and implied potential metabolic activity via changes in adipocyte size and fibrous tissue infiltration. By considering the functions of known fat pads and the results of this study, we speculate multiple functional roles for the IVFP that could be important in IVD health and warrant further investigation.

## AUTHOR CONTRIBUTIONS

**Niklas H. Koehne:** Conceptualization (equal), Data Curation (lead), Formal Analysis (lead), Investigation (lead), Methodology (equal), Validation (lead), Visualization (equal), Writing—Original Draft Preparation (equal). **Irina Heggli:** Conceptualization (equal), Data Curation (supporting), Formal Analysis (equal), Funding Acquisition (supporting), Investigation (supporting), Methodology (equal), Project Administration (supporting), Supervision (lead), Validation (supporting), Visualization Writing—Original Draft Preparation (equal). **Agnieszka Karol:** Investigation (supporting), Methodology (equal), Validation (equal), Writing—Review and Editing (supporting). **Alon Lai:** Resources (lead), Formal Analysis (supporting), Writing—Review and Editing (supporting). **Svenja Illien‐Jünger:** Resources (supporting), Writing—Review and Editing (supporting). **Andrew C. Hecht:** Conceptualization (supporting), Writing—Review and Editing (supporting). **Nilsson Holguin:** Resources (supporting), Writing—Review and Editing (supporting). **James C. Iatridis:** Conceptualization (equal), Funding Acquisition (lead), Methodology (equal), Project Administration (lead), Supervision (supporting), Visualization (supporting), Writing—Original Draft Preparation (equal).

## Supporting information


**Figure S1.** Representative image of a sham intervertebral disc including the annulus fibrosus (AF) and nucleus pulposus (NP), with a full intervertebral fat pad anteriorly.

## Data Availability

The data that support the findings of this study are available from the corresponding author upon reasonable request.
