# Supplementary material for: Newly identified intervertebral fat pad degenerates after intervertebral disc injury in a rat model of degeneration
Source: J Anat. 2025 Dec 14;249(2):435–44. doi: 10.1111/joa.70092 (PMC13339922; doi:10.1111/joa.70092)
Supplement: Supplementary file 1 — Figure S1. Representative image of a sham intervertebral disc including the annulus fibrosus (AF) and nucleus pulposus (NP), with a full intervertebral fat pad anteriorly. [file JOA-249-435-s001.docx]

**Supplemental Figures**

**
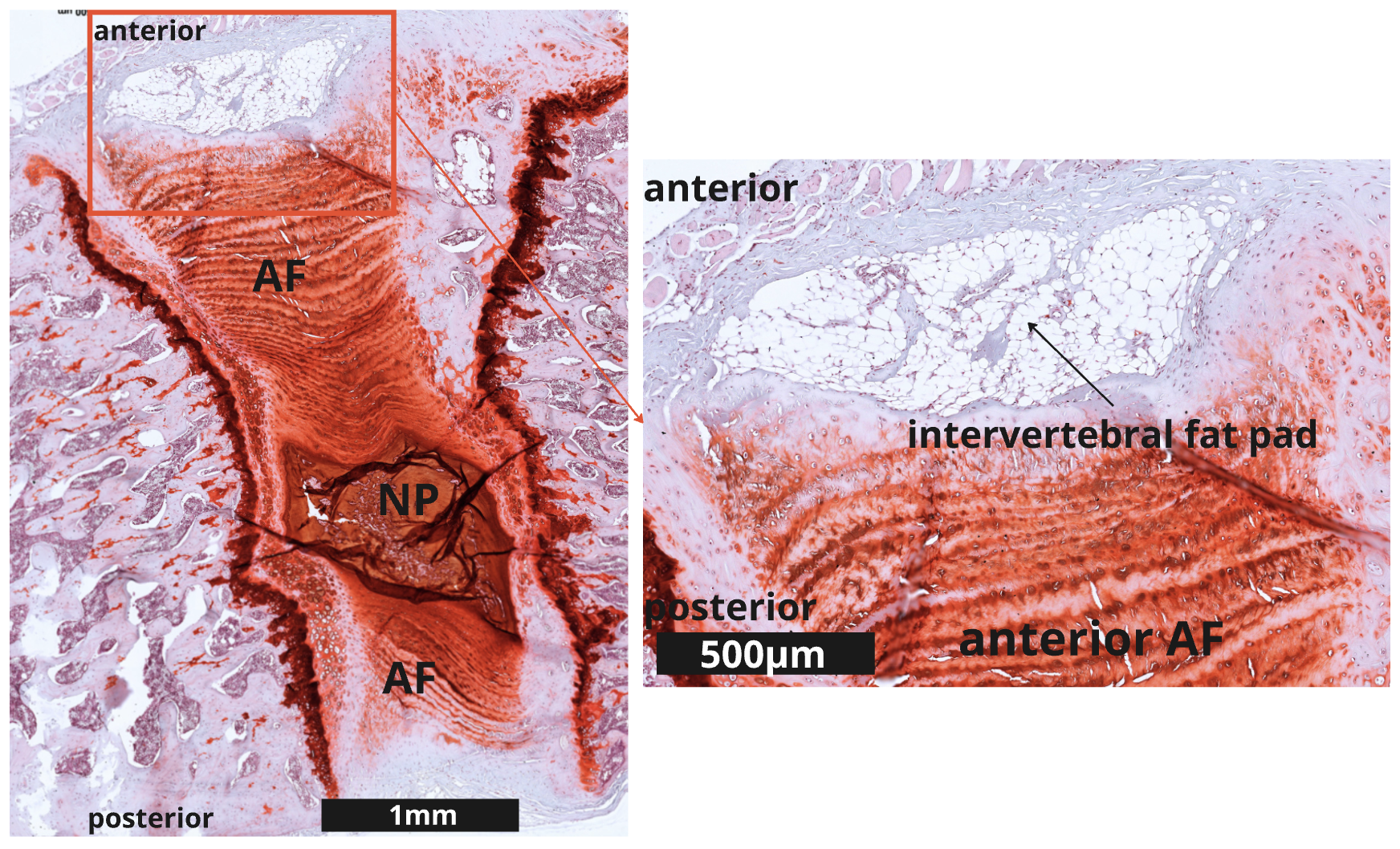
**

**Supplemental Figure 1.** Representative image of a sham intervertebral disc including the annulus fibrosus (AF) and nucleus pulposus (NP), with a full intervertebral fat pad anteriorly.
